# Supplementary material for: The origin and structural evolution of de novo genes in Drosophila
Source: Nat Commun. 2024 Jan 27;15:810. doi: 10.1038/s41467-024-45028-1 (PMC10821953; doi:10.1038/s41467-024-45028-1)
Supplement: Supplementary file 1 — Supplementary Information [file 41467_2024_45028_MOESM1_ESM.pdf]

Supplementary figures for

The origin and structural evolution of *de novo* genes in *Drosophila*

Junhui Peng, Li Zhao
Laboratory of Evolutionary Genetics and Genomics, The Rockefeller University, New York, NY 10065, USA
*Correspondence to: lzhao@rockefeller.edu

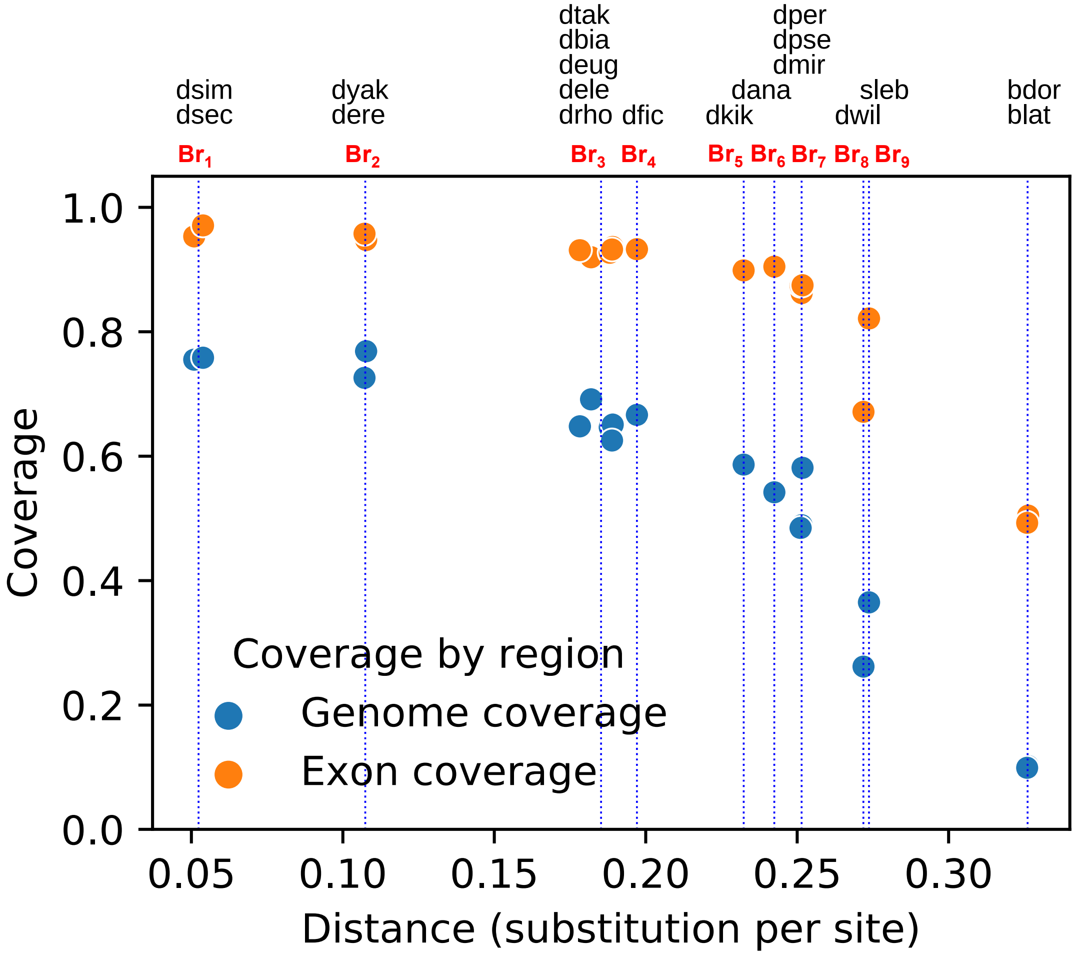

Figure S1. Statistics of progressive cactus alignments.

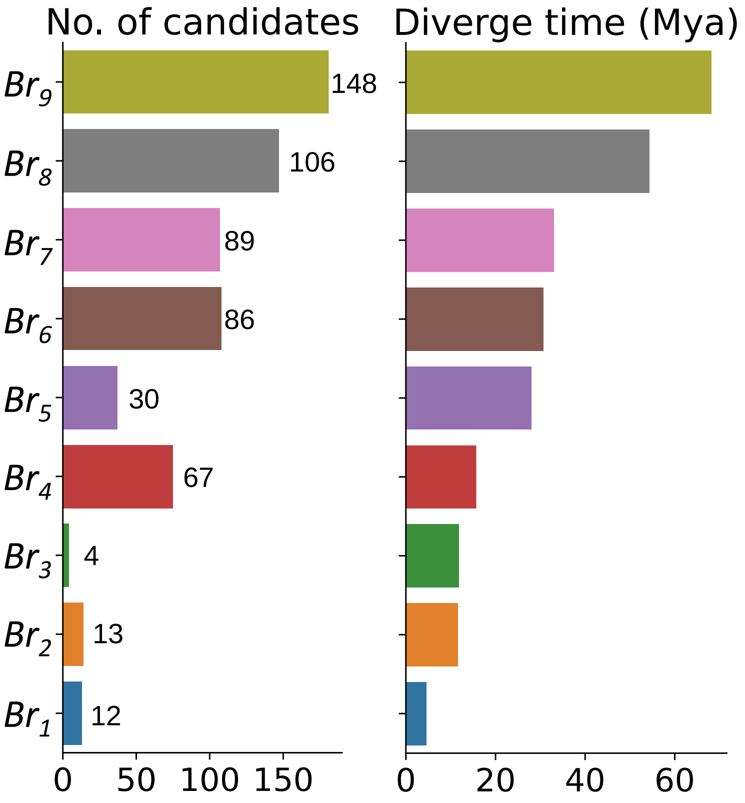

Figure S2. Number of *de novo* gene candidates identified in each branch. The number generally correlated with the divergence time between *D. melanogaster* and each branch.

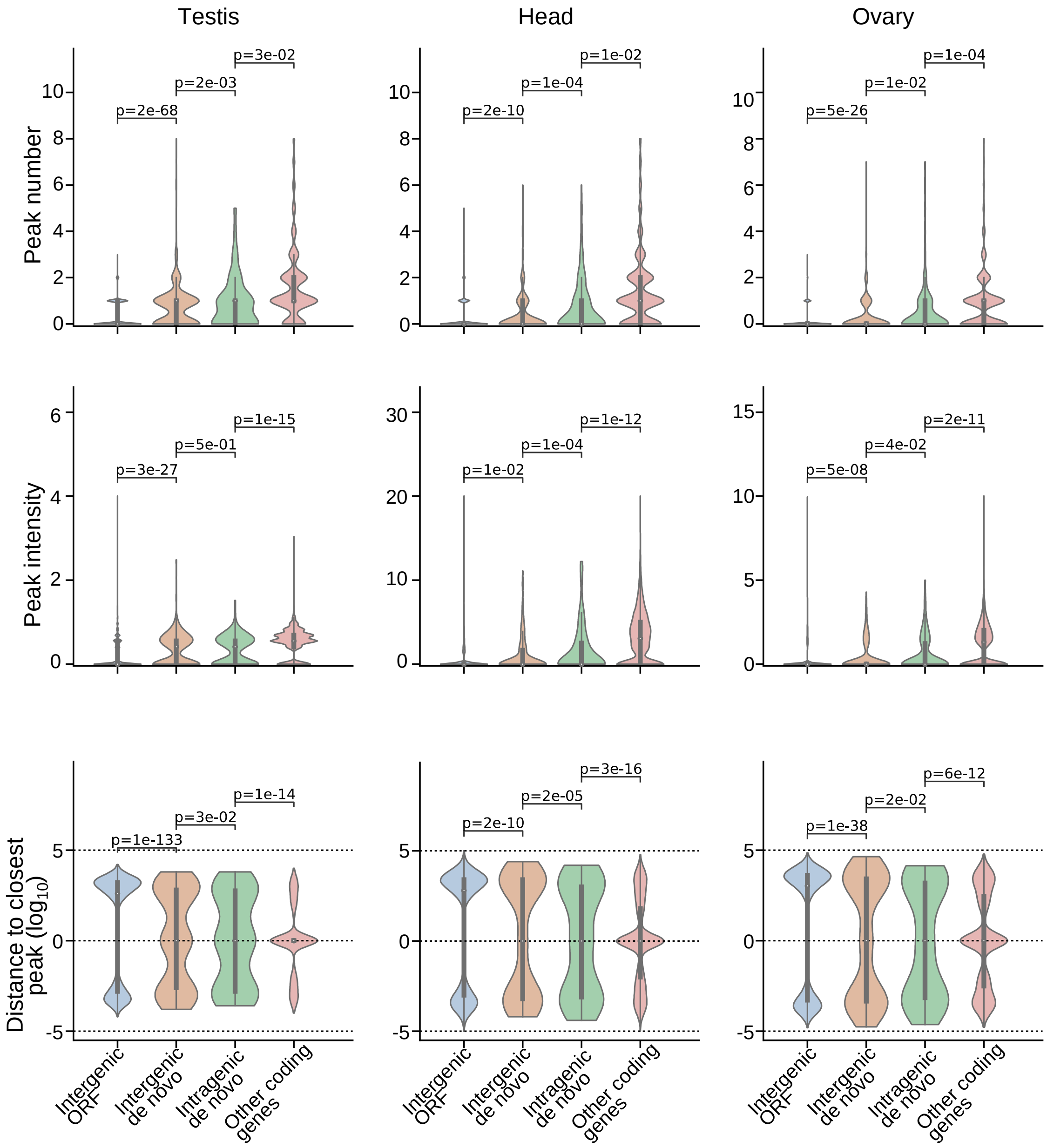

Figure S3. ATAC-seq peaks, intensities, and distances for de novo gene candidates in testis, head, and ovary.

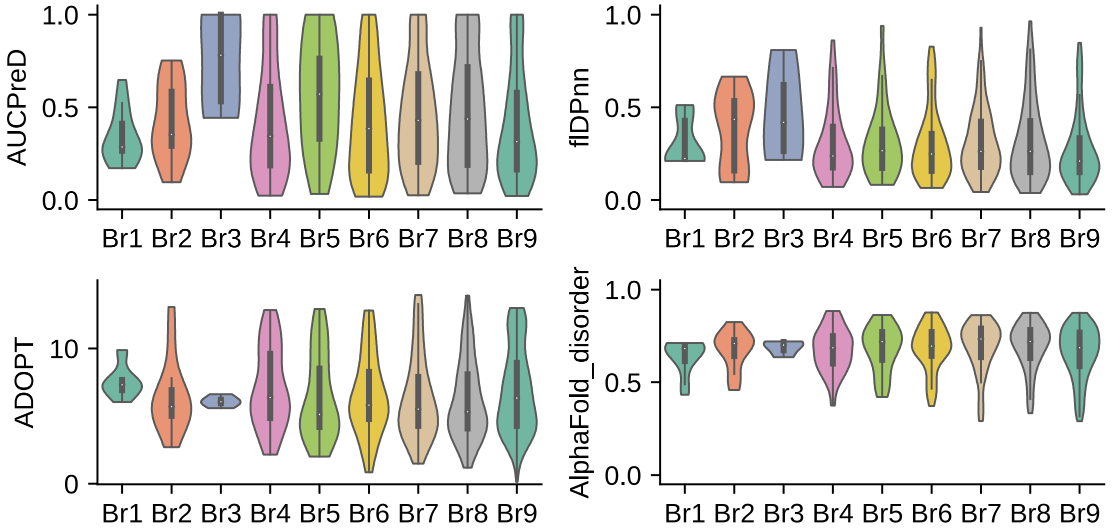

Figure S4. Structural disorder of *de novo* proteins by different state-of-the-art predictors, including AUCPreD (top left panel), flDPnn (top right panel), ADOPT (bottom left panel), and AlphaFold_disorder (bottom right panel). The results overall indicated that the structural disorder of *de novo* proteins changed little with their origination ages.

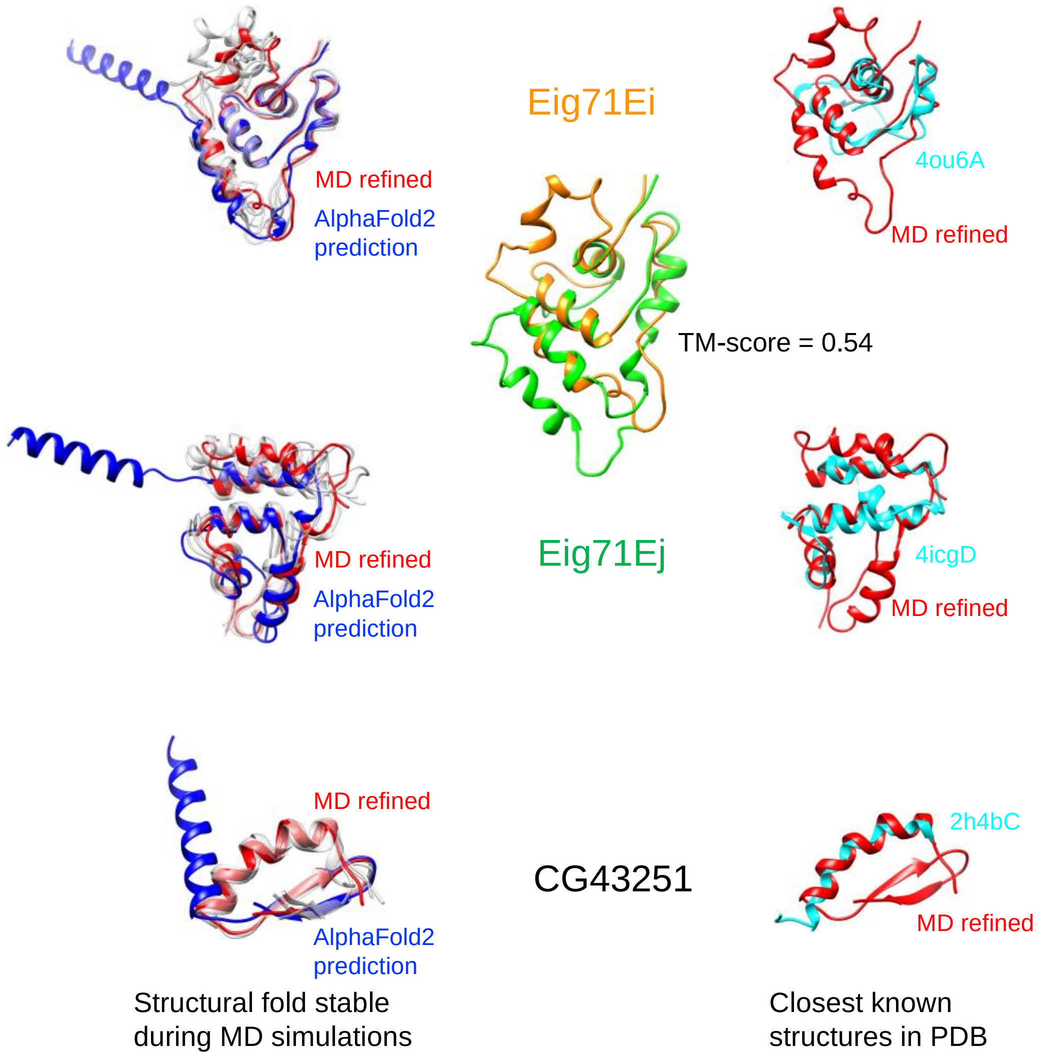

Figure S5. Eig71Ei, Eig71Ej, and CG43251 might adopt new structural folds. Left panel: Eig71Ei, Eig71Ej, and CG43251 remained similar structural folds during MD simulations. Right panel: the structures in PDB with the largest TM-scores to Eig71Ei, Eig71Ej, and CG43251 were superimposed to their MD refined structural models. All the three largest TM-scores were smaller than 0.5 (Table S1). Eig71Ei and Eig71Ej are paralogs and share similar structural folds with TM-score of 0.54 (inserted panel).

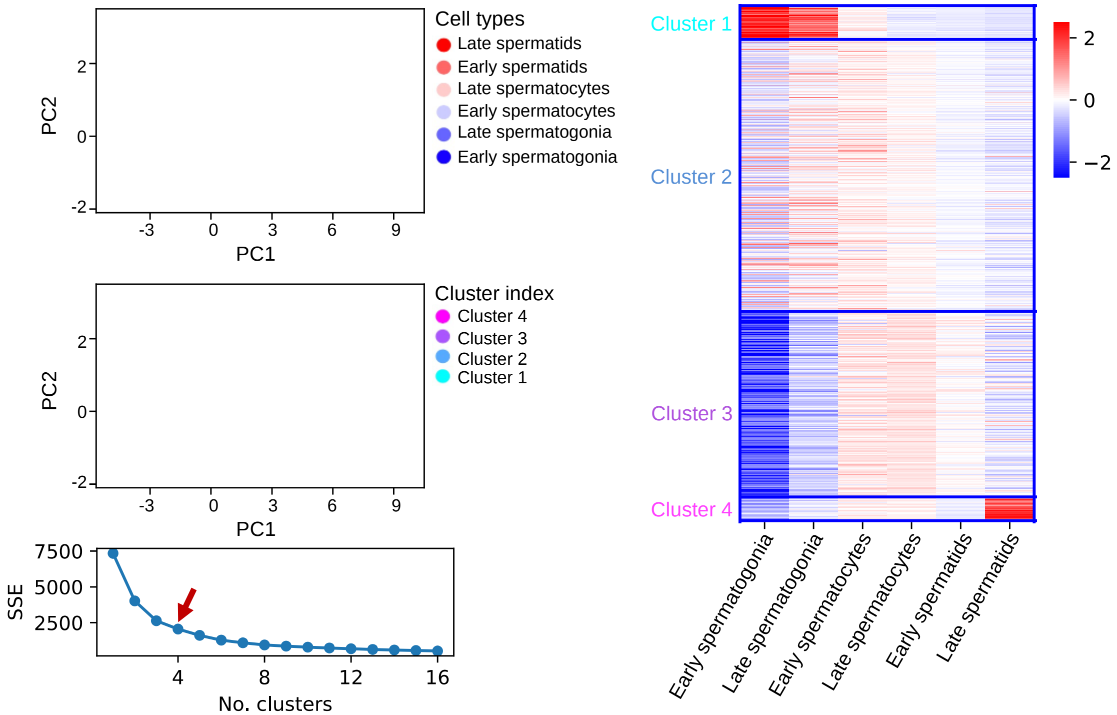

Figure S6. Clustering of all *D. melanogaster* testis-biased genes. The sum of squared error (SSE) as a function of the number of clusters was shown in the bottom left panel.

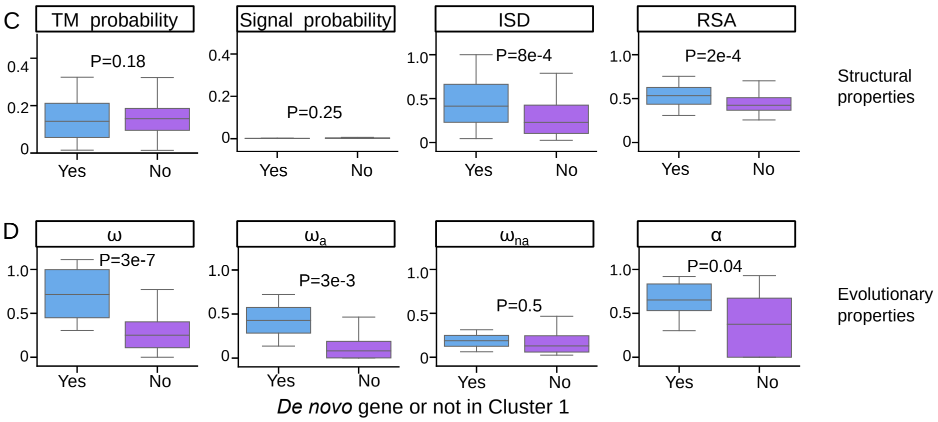

Figure S7. Comparison of testis-biased *de novo* genes (cyan) and non-*de novo* genes (purple) in cluster #1. For clustering analysis, see Figure 5 for detail. In cluster #1, testis biased de novo genes are more disordered (ISD panel) and exposed (RSA panel). These de novo genes also evolve faster (ω panel) with higher adaptation rates (ω_a panel).

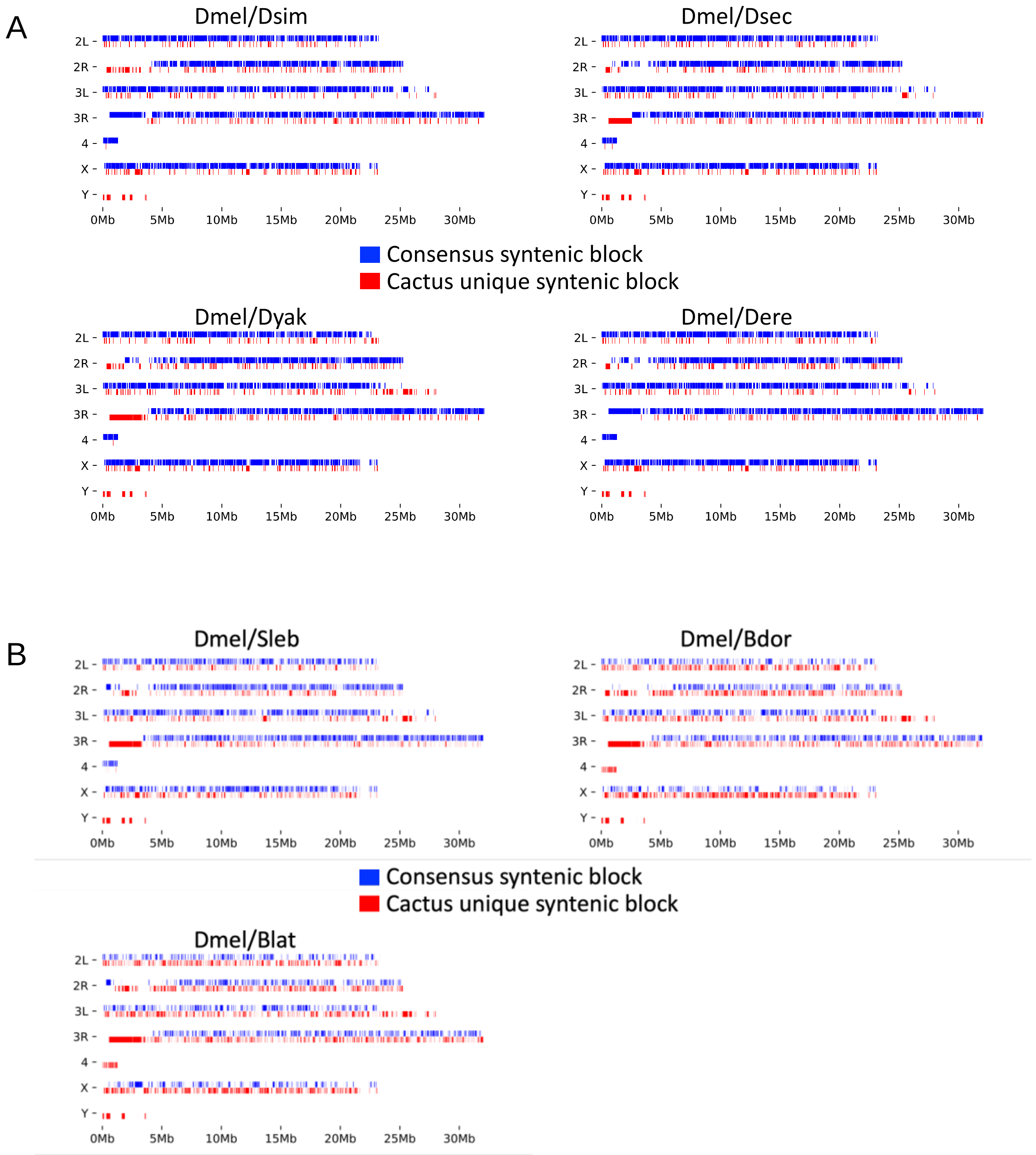

Figure S8. Comparison of synteny blocks recovered by cactus aligner and micro-synteny method in (A) four closely related genomes (*D. simulans*, *D. sechellia*, *D. yakuba*, *and D. erecta*), and (B) three distantly related genomes (*S. lebanonensis*, *B. dorsalis*, and *B. latifrons*). The figure shows that Cactus aligner recover more syntenic regions.

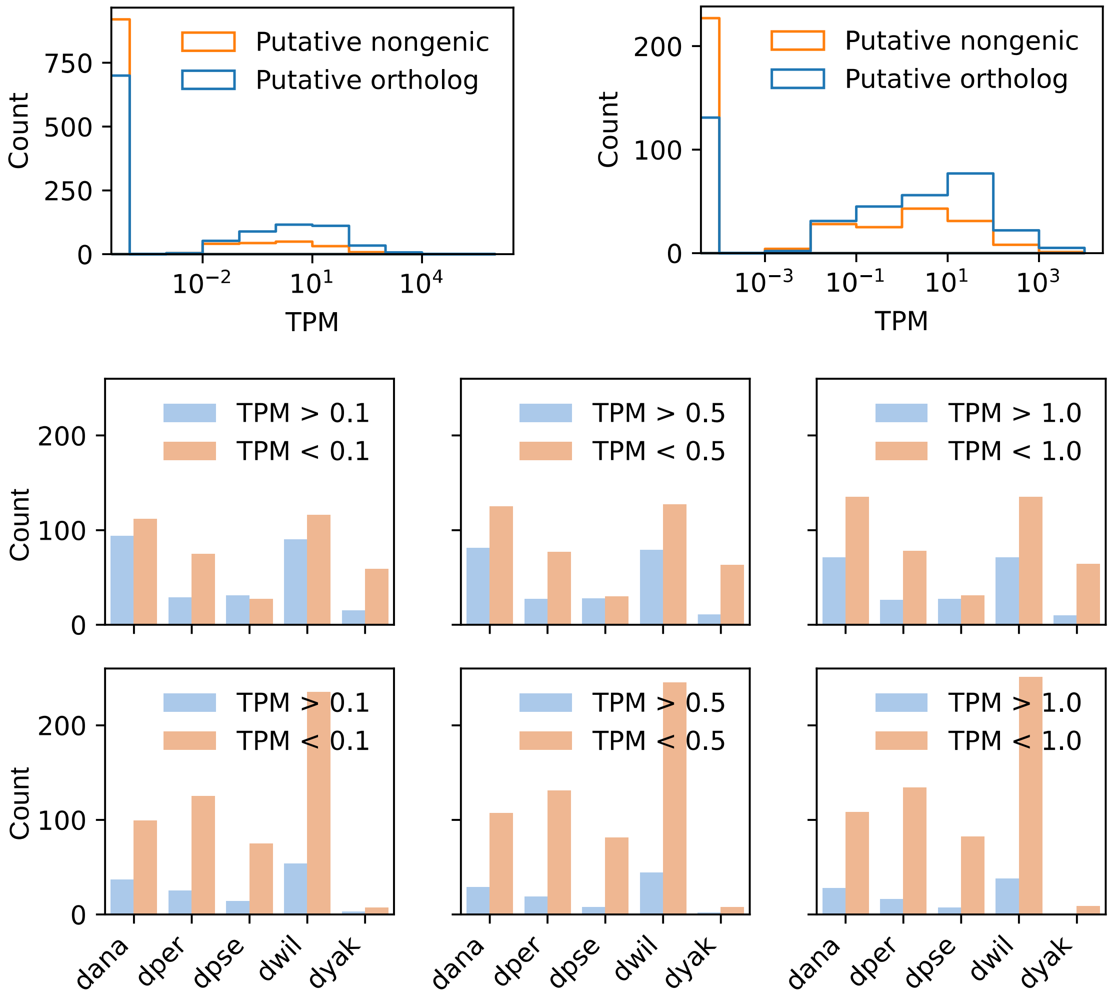

Figure S9. RNA-seq support of the unannotated putative orthologs of some de novo gene candidates in *D. yakuba*, *D. ananassae*, *D. persimilis*, *D. pseudoobscura*, *D. willistoni*.

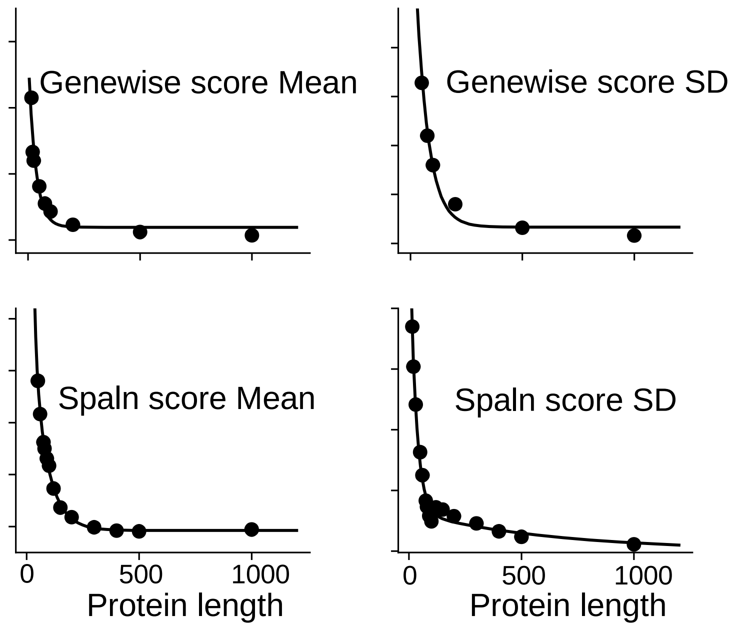

Figure S10. Random simulations of Genewise/Spaln. We used two-phase decay function to fit the mean (left panel) and standard error (right panel) of spliced align score (Genewise, top panel, and Spaln, bottom panel).

Table S1. Proportions of optimal codons in *de novo* genes and other annotated protein-coding genes in *D. melanogaster*. The median values of the proportions were listed in the P(optimal, *de novo*) and P(optimal, other) columns. *De novo* genes show significantly less optimal codon usage compared to other annotated protein-coding genes. The P-values were computed using *scipy.stats.ttest_ind* module with the option one-sided test, *alternative="less"*, and were shown in the P(t-test) column. Most amino acids show a significant positive correlation between the proportion of optimal codons and the origination branches, as shown by the P-values of the two-sided Spearman and Kendall tau rank correlation tests.
.

Amino Acid	P(optimal, *de novo*)	P(optimal, other)	P(t-test)	P(Spearman r)	P(Kendall tau)
A	0.32	0.45	9.2E-70	4.4E-07	5.3E-07
C	0.62	0.73	3.1E-25	5.9E-02	5.8E-02
D	0.38	0.46	1.3E-16	5.8E-01	5.6E-01
E	0.50	0.68	7.1E-95	6E-07	9.9E-07
F	0.50	0.63	1.6E-18	2.5E-03	2.6E-03
G	0.28	0.42	2.2E-59	2.2E-07	2.2E-07
H	0.50	0.60	5.2E-31	3E-04	3.8E-04
I	0.33	0.48	2.6E-22	1.7E-03	1.9E-03
K	0.53	0.71	1.5E-52	1.2E-01	1.2E-01
L	0.25	0.42	5.6E-94	4.5E-04	4.5E-04
M	1.00	1.00	nan	nan	nan
N	0.50	0.55	5.6E-14	3.7E-01	3.9E-01
P	0.23	0.33	3E-40	2.6E-03	3.1E-03
Q	0.50	0.71	8.2E-71	5.9E-03	5.9E-03
R	0.14	0.30	6.8E-70	5E-07	9.7E-07
S	0.19	0.24	1.5E-18	1.8E-02	1.7E-02
T	0.25	0.38	1.8E-38	5E-05	5.7E-05
V	0.33	0.47	3.9E-70	3.2E-04	3.7E-04
W	1.00	1.00	nan	nan	nan
Y	0.50	0.64	1.3E-09	1.5E-02	1.4E-02

Table S2. Detailed information of potentially well folded *de novo* gene candidates. Candidates with TM-score to structures in PDB smaller than 0.5 were highlighted in red. A TM-score lower than 0.5 suggests a possible novel structural fold.

FBID	pLDDT	Length	Origination lineage	pLDDT (Anc)	TM-score (ToPDB)	Similar fold in PDB	Sequence identity	Gene
**FBgn0004593**	0.89	98	Br_7	0.92	0.40	4ou6A	0.087	Eig71Ef
**FBgn0014850**	0.92	98	Br_7	0.92	0.45	4icgD	0.053	Eig71Ej
**FBgn0262896**	0.80	39	Br_5	0.70	0.49	2h4bC	0.158	CG43251
**FBgn0260967**	0.90	280	Br_8	0.91	0.56	6xgxB	0.075	CG42590
**FBgn0265834**	0.85	153	Br_4	0.84	0.68	1u89A	0.066	CG44623
**FBgn0261580**	0.88	137	Br_6	0.78	0.60	4pr9F	0.066	CG42690
**FBgn0261587**	0.86	139	Br_7	0.86	0.59	5fjeB	0.022	CG42697
**FBgn0263250**	0.87	127	Br_3	0.88	0.61	1x91A	0.023	CG43393
**FBgn0261581**	0.85	140	Br_6	0.78	0.61	6q6bD	0.028	CG42691
**FBgn0262819**	0.91	114	Br_6	0.77	0.62	5figF	0.062	CG43190
**FBgn0265046**	0.91	118	Br_6	0.81	0.64	5fjdB	0.076	CG44163
**FBgn0052192**	0.87	136	Br_3	0.86	0.64	5fjdB	0.074	CG32192
**FBgn0037042**	0.93	195	Br_8	0.92	0.65	7jh6B	0.093	CG12984
**FBgn0264748**	0.93	374	Br_6	0.90	0.66	1yrgB	0.096	CG44006
**FBgn0264747**	0.92	370	Br_6	0.90	0.66	1yrgB	0.138	CG44005
**FBgn0264746**	0.92	368	Br_6	0.90	0.67	6obnC	0.133	CG44004
**FBgn0262480**	0.89	126	Br_5	0.67	0.68	6q58D	0.064	CG43070
**FBgn0262824**	0.85	138	Br_6	0.78	0.70	1u89A	0.044	CG43195
**FBgn0263647**	0.92	122	Br_5	0.91	0.76	5me8A	0.057	CG43638

Table S3. MD simulations of 19 potentially well-folded de novo gene candidates. Details of the calculation of structural similarity during MD simulations can be found in Material and Methods.

FBID	Name	RMSD_FL	RMSD_CORE	TM-score
**FBgn0037042**	CG12984	1.95	1.88	0.91
**FBgn0264748**	CG44006	1.73	1.67	0.95
**FBgn0264747**	CG44005	2.22	1.87	0.94
**FBgn0014850**	Eig71Ej	4.53	1.90	0.76
**FBgn0263647**	CG43638	1.22	1.18	0.94
**FBgn0264746**	CG44004	2.58	1.74	0.94
**FBgn0262819**	CG43190	2.87	2.08	0.83
**FBgn0265046**	CG44163	1.70	1.27	0.92
**FBgn0260967**	CG42590	1.47	1.47	0.96
**FBgn0004593**	Eig71Ef	5.55	2.51	0.69
**FBgn0262480**	CG43070	1.41	1.24	0.92
**FBgn0261580**	CG42690	3.67	2.68	0.77
**FBgn0052192**	CG32192	3.98	2.76	0.76
**FBgn0263250**	CG43393	2.09	1.69	0.87
**FBgn0261587**	CG42697	2.29	2.07	0.86
**FBgn0265834**	CG44623	4.33	2.37	0.79
**FBgn0261581**	CG42691	4.94	3.07	0.71
**FBgn0262824**	CG43195	3.33	2.39	0.81
**FBgn0262896**	CG43251	2.84	1.85	0.68

Table S4. Number of *D. melanogaster* protein-coding gene orthologs recovered by orthoMCL, Cactus aligner, and MCscanX with micro-synteny option. The number of overlaps between Cactus and orthoMCL, and MCScanX and orthoMCL are shown in parenthesis.

**species**	**orthoMCL**	**Cactus**	**MCScanX(Micro-synteny)**
Dsim	13486	13584 (10717)	11726 (9870)
Dsec	13427	13624 (11498)	11688 (10582)
Dyak	13229	13429 (10414)	11361 (9245)
Dere	13330	13507 (11439)	11645 (10332)
Dfic	12860	13257 (10868)	11107 (9496)
Drho	12963	13255 (10330)	9241 (7880)
Dele	12860	13242 (10863)	10894 (9416)
Deug	13008	13314 (10874)	11187 (9656)
Dtak	13124	13385 (11048)	10757 (9346)
Dbia	13036	13326 (11087)	11163 (9670)
Dkik	12539	12902 (10642)	10088 (8666)
Dana	12579	12993 (10764)	10538 (9049)
Dper	12138	12592 (10376)	10023 (8427)
Dpse	12174	12639 (10513)	10055 (8565)
Dmir	12193	12635 (10192)	10144 (8250)
Dwil	11795	12205 (9951)	9057 (7449)
Sleb	11483	10401 (8502)	8968 (7455)
Blat	9774	8737 (6822)	4861 (3778)
Bdor	9684	8684 (5785)	4840 (3254)
