# Supplementary material for: The origin and structural evolution of de novo genes in Drosophila
Source: Nat Commun. 2024 Jan 27;15:810. doi: 10.1038/s41467-024-45028-1 (PMC10821953; doi:10.1038/s41467-024-45028-1)
Supplement: Supplementary file 3 — Description of Additional Supplementary Files [file 41467_2024_45028_MOESM3_ESM.pdf]

Description of Additional Supplementary Files

File Name: Supplementary Data 1

Description: This data includes four sections detailing genome versions, accession codes, and alignments of 1285 potential candidates with non-genic hits in whole genome alignments within Br9. Section I includes the versions and accession codes of the genomes used in this study. Section II includes raw alignments of 1285 potential candidates with non-genic hits in whole genome alignments within Br9. Section III includes spliced alignments by spaln. Section IV comprises spliced alignments by genewise.

File Name: Supplementary Data 2

Description: The FlyBase ID and properties of the 555 *de novo* gene candidates identified in this study.

File Name: Supplementary Data 3

Description: Structural dynamics of the ancestral structures of potentially well-folded *de novo* proteins in MD simulations.

File Name: Supplementary Data 4

Description: Filtered candidates with homologs in jackhmmer and blastp search against UniprotKB database and tblastn search against NCBI representative genomes.

File Name: Supplementary Data 5

Description: The full list of the 13968 annotated protein-coding genes in *D. melanogaster* (FlyBase version 6). The data shows whether the genes were among 13798 aligned in Cactus, 1285 potential candidates with non-genic hits, 686 preliminary candidates, or final 555 de novo gene candidates.
