# Supplementary material for: The origin and structural evolution of de novo genes in Drosophila
Source: Nat Commun. 2024 Jan 27;15:810. doi: 10.1038/s41467-024-45028-1 (PMC10821953; doi:10.1038/s41467-024-45028-1)
Supplement: Supplementary file 9 — Reporting Summary [file 41467_2024_45028_MOESM9_ESM.pdf]

nature portfolio

Corresponding author(s): Li Zhao

Last updated by author(s): Mar 1, 2023

Reporting Summary

Nature Portfolio wishes to improve the reproducibility of the work that we publish. This form provides structure for consistency and transparency in reporting. For further information on Nature Portfolio policies, see our Editorial Policies and the Editorial Policy Checklist.

Statistics

For all statistical analyses, confirm that the following items are present in the figure legend, table legend, main text, or Methods section.

n/a	Confirmed	
☐	☒	The exact sample size (n) for each experimental group/condition, given as a discrete number and unit of measurement
☒	☐	A statement on whether measurements were taken from distinct samples or whether the same sample was measured repeatedly
☐	☒	The statistical test(s) used AND whether they are one- or two-sided<br>*Only common tests should be described solely by name; describe more complex techniques in the Methods section.*
☒	☐	A description of all covariates tested
☐	☒	A description of any assumptions or corrections, such as tests of normality and adjustment for multiple comparisons
☐	☒	A full description of the statistical parameters including central tendency (e.g. means) or other basic estimates (e.g. regression coefficient) AND variation (e.g. standard deviation) or associated estimates of uncertainty (e.g. confidence intervals)
☐	☒	For null hypothesis testing, the test statistic (e.g. F, t, r) with confidence intervals, effect sizes, degrees of freedom and P value noted<br>*Give P values as exact values whenever suitable.*
☒	☐	For Bayesian analysis, information on the choice of priors and Markov chain Monte Carlo settings
☒	☐	For hierarchical and complex designs, identification of the appropriate level for tests and full reporting of outcomes
☐	☒	Estimates of effect sizes (e.g. Cohen's d, Pearson's r), indicating how they were calculated

Our web collection on statistics for biologists contains articles on many of the points above.

Software and code

Policy information about availability of computer code

Data collection	No software or code needed for data collection.
Data analysis	CACTUS 1.2.3; halLiftover 2.1; BLAST 2.10.1+; HMMER 3.3.1; Genewise 2.4.1; Spaln 2.4.4; MAFFT v7.429; AlphaFold v2.0; LMFIT 1.0.3; RepeatMasker 4.1.0; Dfam 3.2; ORFfinder, last accessed 05/04/2021; DeepCNF 1.0.2; SignalP v5.0; RosettaFold v1.0.0; trRosetta Version 1; TM-align Version 20210520; GROMACS Version 2019.1; scikit-learn 1.0.2; esmfold v1; fldpnn; ADOPT; AlphaFold-disorder

For manuscripts utilizing custom algorithms or software that are central to the research but not yet described in published literature, software must be made available to editors and reviewers. We strongly encourage code deposition in a community repository (e.g. GitHub). See the Nature Portfolio guidelines for submitting code & software for further information.

Data

Policy information about availability of data

All manuscripts must include a data availability statement. This statement should provide the following information, where applicable:

- Accession codes, unique identifiers, or web links for publicly available datasets
- A description of any restrictions on data availability
- For clinical datasets or third party data, please ensure that the statement adheres to our policy

Whole genome alignments used in this study are available at 10.6084/m9.figshare.19989395, along with the raw alignments and spliced alignments of de novo gene

candidates, which include noncoding hits in outgroup species. Code and scripts for this study are available on GitHub: https://github.com/LiZhaoLab/DrosophilaDenovoGene as well as on Zenodo: https://doi.org/10.5281/zenodo.10233859

Human research participants

Policy information about studies involving human research participants and Sex and Gender in Research.

Reporting on sex and gender	N/A
Population characteristics	N/A
Recruitment	N/A
Ethics oversight	N/A

Note that full information on the approval of the study protocol must also be provided in the manuscript.

Field-specific reporting

Please select the one below that is the best fit for your research. If you are not sure, read the appropriate sections before making your selection.

☐ Life sciences ☐ Behavioural & social sciences ☒ Ecological, evolutionary & environmental sciences

For a reference copy of the document with all sections, see nature.com/documents/nr-reporting-summary-flat.pdf

Ecological, evolutionary & environmental sciences study design

All studies must disclose on these points even when the disclosure is negative.

Study description	In this study, we applied highly accurate reference-free progressive whole genome alignments to identify 555 protein-coding de novo gene candidates in Drosophilinae lineage. We then studied their evolutionary and structural properties. In addition to revealing the patterns and properties of their secondary structures, we combined AlphaFold2 prediction and MD simulations to study the structures and structural evolution of the identified de novo gene candidates.
Research sample	The samples are the 555 de novo genes identified through our pipeline, which is a main focus of our study
Sampling strategy	N/A
Data collection	N/A
Timing and spatial scale	N/A
Data exclusions	Outliers in the boxplots are neglected when plotting the figures. However, all data points were used for statistical analyses.
Reproducibility	Whole genome alignments used in this study are available at 10.6084/m9.figshare.19989395, with the alignments of de novo gene candidates, which include noncoding hits in outgroup species. Code and scripts for this study are available on GitHub: https://github.com/LiZhaoLab/DrosophilaDenovoGene as we as on Zenodo: https://doi.org/10.5281/zenodo.10233859
Randomization	The null of certain properties, such as the random expectation for the genewise/spaln gene structure, were generated by the python random library.
Blinding	Blinding is not needed for this work.

Did the study involve field work? ☐ Yes ☒ No

Reporting for specific materials, systems and methods

We require information from authors about some types of materials, experimental systems and methods used in many studies. Here, indicate whether each material, system or method listed is relevant to your study. If you are not sure if a list item applies to your research, read the appropriate section before selecting a response.

Materials & experimental systems

n/a	Involved in the study
☒	☐ Antibodies
☒	☐ Eukaryotic cell lines
☒	☐ Palaeontology and archaeology
☒	☐ Animals and other organisms
☒	☐ Clinical data
☒	☐ Dual use research of concern

Methods

n/a	Involved in the study
☒	☐ ChIP-seq
☒	☐ Flow cytometry
☒	☐ MRI-based neuroimaging
